# Key Issues in the Role of Peroxisome Proliferator–Activated Receptor Agonism and Cell Signaling in Trichloroethylene Toxicity

**DOI:** 10.1289/ehp.8693

**Published:** 2006-05-09

**Authors:** Nagalakshmi Keshava, Jane C. Caldwell

**Affiliations:** National Center for Environmental Assessment, Office of Research and Development, U.S. Environmental Protection Agency, Washington, DC, USA

**Keywords:** dichloroacetic acid, peroxisome proliferator-activated receptor, PPAR, trichloroacetic acid, trichloroethylene

## Abstract

Peroxisome proliferator–activated receptor α (PPARα) is thought to be involved in several different diseases, toxic responses, and receptor pathways. The U.S. Environmental Protection Agency 2001 draft trichloroethylene (TCE) risk assessment concluded that although PPAR may play a role in liver tumor induction, the role of its activation and the sequence of subsequent events important to tumorigenesis are not well defined, particularly because of uncertainties concerning the extraperoxisomal effects. In this article, which is part of a mini-monograph on key issues in the health risk assessment of TCE, we summarize some of the scientific literature published since that time on the effects and actions of PPARα that help inform and illustrate the key scientific questions relevant to TCE risk assessment. Recent analyses of the role of PPARα in gene expression changes caused by TCE and its metabolites provide only limited data for comparison with other PPARα agonists, particularly given the difficulties in interpreting results involving *PPAR*α knockout mice. Moreover, the increase in data over the last 5 years from the broader literature on PPARα agonists presents a more complex array of extraperoxisomal effects and actions, suggesting the possibility that PPARα may be involved in modes of action (MOAs) not only for liver tumors but also for other effects of TCE and its metabolites. In summary, recent studies support the conclusion that determinations of the human relevance and susceptibility to PPARα-related MOA(s) of TCE-induced effects cannot rely on inferences regarding peroxisome proliferation per se and require a better understanding of the interplay of extraperoxisomal events after PPARα agonism.

Trichloroethylene (TCE) and its metabolites trichloroacetic acid (TCA) and dichloroacetic acid (DCA) induce peroxisome proliferation (PP) in rodents; only TCA and DCA activate mouse and human PP-activated receptor α (PPARα) *in vitro*, and TCA induces the most sustained PP response (Bull 2000; Maloney and Waxman 1999; Zhou and Waxman 1998). However, all three are relatively weak inducers of PP compared with the pharmaceutical drug Wyeth-14,463 (WY), which is considered to be the “model” agonist of PPARα and thought to be responsible for PP. Modes of action (MOAs) for TCE involving PP or PPARα agonism generally have focused on induction of liver tumors, for which associations with TCE and/or its metabolites have been reported in both rodent bioassays and human epidemiologic studies [U.S. Environmental Protection Agency (U.S. EPA) 2001; Wartenberg et al. 2000]. PPAR-independent MOAs of TCE metabolites (e.g., inhibition of glutathione *S*-transferase ζ by DCA or hypomethylation by TCA or DCA) are discussed separately in Caldwell and Keshava (2006).

There are a number of both long-standing and emerging issues with respect to evaluating the role of PPARα in MOAs for TCE toxicity. The U.S. EPA draft TCE risk assessment (U.S. EPA 2001) concluded that although PPARα may play a role in liver tumor induction, the role of its activation in the sequence of events leading to tumorigenesis was not well defined, particularly due to uncertainties in the contribution and cross-species relevance of extra-peroxisomal effects from PPARα activation. Moreover, a vast literature on PPARα agonists has emerged investigating its potential role not only in liver tumorigenesis but also in numerous other diseases, toxic responses, and receptor pathways. This suggests that investigation of possible roles of PPARα agonism in the MOAs of TCE toxicity should move beyond examining only liver tumorigenesis.

In the present article we highlight some of the recently published literature on PPARα for TCE, its metabolites, and other PPARα agonists to help inform and illustrate the key scientific issues relevant to TCE risk assessment. Although some scientific conclusions can be drawn from this updated body of data, speculation as to its impact on the final TCE risk assessment would be premature at this point, given the ongoing National Academy of Sciences consultation discussed in the overview article (Chiu et al. 2006) and the subsequently planned revision of the U.S. EPA TCE risk assessment. Therefore, the purpose here and throughout this mini-monograph is to provide a review of recently published scientific literature in the context of how it informs the key scientific issues we believe to be most critical to developing a revised risk assessment.

## Recent Data on PPARα Agonism and TCE

Recent efforts to elucidate the role of PPARα agonism in TCE-induced toxicity have focused on comparison of gene expression changes with other agonists and/or the use of *PPAR* α knockout mice. Recent data on DNA methylation changes and other MOAs for a number of agonists, including TCE and its metabolites DCA and TCA, are reported elsewhere in this mini-monograph (Caldwell and Keshava 2006). TCE-specific data using DNA arrays and knockout mice remain limited and difficult to interpret.

It is difficult to discern a clear pattern of common gene expression changes among TCE and its metabolites or for peroxisome proliferators in general for use in making inferences regarding common MOAs. For example, in a screening analysis of 148 genes for xenobiotic-metabolizing enzymes, DNA repair enzymes, heat-shock proteins (hsp), cytokines, and housekeeping genes in mouse liver, Bartosiewicz et al. (2001b) reported TCE-induced up-regulation of only three genes [*hsp25* and *hsp86*, and cytochrome P450 2a (*cyp2a*)] at the highest dose tested (1,000 mg/kg) and repression of *cyp2a* at a much lower single dose (10 mg/kg) of TCE after a single intraperitoneal injection in corn oil. Using a similar paradigm with 260 genes, Bartosiewicz et al. (2001a) reported that exposure to 500 mg/kg clofibrate and 1,100 mg/kg di(2-ethylhexyl)phthalate (DEHP) induced a different pattern of transcription than did TCE. DEHP and clofibrate cause increases in gene expression of acyl–coenzyme A (CoA) thioesterase, *cyp4a10*, and insulin-like growth factor (IGF), with clofibrate also inducing greater expression of these genes and additional induction of *cyp2b9*, a fatty acid–binding protein, and metallothionein II. The pattern of induction differed between kidney and liver for DEHP and clofibrate. Collier et al. (2003) reported 26 differentially expressed mRNA transcripts in embryonic hearts of Sprague-Dawley rats whose dams were exposed to 1,100 ppm TCE through drinking water between days 0 and 11 of pregnancy. Genes down-regulated with TCE exposure appear to be those associated with cellular housekeeping, cell adhesion, and developmental processes, whereas TCE exposure up-regulated expression of numerous stress-response and homeostatic genes.

Two studies have used *PPAR*α knockout mice to investigate the importance of PPARα to TCE toxicity. However, interpretation of *PPAR*α knockout mice data in general poses some unique difficulties due to differences in baseline responses, some of which were observed in the TCE-specific studies as well. In one study, Laughter et al. (2004) used macroarrays containing approximately 1,200 genes and reported altered expression of 43 genes in the TCE-treated wild-type mice and 67 genes in *PPAR*α knockout mice after 3 days of exposure to up to 1,500 mg/kg/day TCE. The authors reported that of the 43 genes with altered expression in wild-type mice after TCE exposure, 40 genes were dependent on *PPAR* α. These genes included *cyp4a12*, epidermal growth factor receptor, and additional genes involved in cell growth. However, the interpretation of this information is difficult because a comparison of gene expression profiles between controls (wild-type and *PPAR*α knockout) was not reported. Moreover, after 3 weeks of TCE treatment (0–1,500 mg/kg via gavage), Laughter et al. (2004) reported toxicity at the 1,500 mg/kg level in the knockout mice that was not observed in the wild-type mice; all knockout mice were moribund and had to be removed from the study. Inspections of livers and kidneys from the group did not reveal overt signs of toxicity that would lead to morbidity. At the same dose, wild-type mice exhibited mild granuloma formation with calcification or mild hepatocyte degeneration with centrilobular hepatocyte hypertrophy. A TCE treatment–related increase in liver weight was reported in wild-type mice but not in knockout mice. However, knockout mice had a greater liver-to-body weight ratio than did wild-type mice at all levels of exposure, including controls, making detection of a TCE-induced change difficult. Similarly, the knockout mice also had higher baseline levels of hepatocyte proliferation. Both knockout and wild-type mice appeared to have similar levels of hepatocyte proliferation after 1,000 mg/kg TCE, with a high variability in response. No analysis was reported to determine a statistical difference in proliferation between the two types of mice as a consequence of TCE exposure. Kidney-to-body weight ratios were increased in wild-type but not in knockout mice compared with controls. No changes in kidney weights were reported after 3 weeks of exposure.

In an earlier study, Nakajima et al. (2000) reported that the number of peroxisomes in hepatocytes increased by 2-fold in wild-type mice but not in *PPAR*α knockout mice after 2 weeks of TCE exposure by gavage (0.75 g/kg). However, TCE induced increased liver weight in both male and female wild-type and knockout mice, suggesting hepatic effects independent of PPARα activation. Interestingly, Laughter et al. (2004) reported no difference in liver-to-body weight ratios between wild-type and knockout mice after 1 week of exposure to 2.0 g/L TCA and only a small difference after 1 week of 2.0 g/L DCA. The authors suggested liver weight changes as a surrogate for peroxisomal proliferative activity, although neither PP nor changes in glycogen content (which also can affect weight) of the liver were directly measured.

## MOAs for Liver Toxicity

Klaunig et al. (2003) proposed an MOA for liver carcinogenicity in rodents of PPARα activation, associated PP, increased cell proliferation, decreased apoptosis, and clonal expansion of preneoplastic cells, but there are notable inconsistencies with this hypothesis. Long-term carcinogenicity studies of the PPARα agonist gemfibrozil (GEM) showed a dose-related increase in liver tumors in male rats, whereas in females a dose-dependent decrease in liver tumors was reported (International Agency for Research on Cancer 1996). Klaunig et al. (2003) place substantial weight on PP as an associative event in their proposed MOA, viewing PP as an indicator of sensitivity to hepatocarcinogenic effects. However, studies in rats with two PPARα agonists, WY and DEHP, demonstrated that doses that produced equivalent levels of hepatic PP, measured as peroxisome number and peroxisomal enzyme activity, produced markedly different liver tumor incidences. The degree of PP correlated poorly with the relative hepatocarcinogenicity of DEHP and WY but was correlated with the ability to induce a persistent increase in replicative DNA synthesis (Marsman et al. 1988).

In another study Reddy and Rao (1989) hypothesized MOA is DNA damage caused by marked increases in free radical–generating enzymes of the peroxisomal β-oxidation through hydrogen peroxide. However, Bannasch (1996) noted that this hypothesis is not supported by the findings in rats treated with the peroxisome proliferator dehydroepiandrosterone, a potential natural regulator of the peroxisomal compartment. Amphophilic cell foci preceding the appearance of hepatocellular neoplasms do not develop from the perivenular zones, in which the most pronounced PP occurs but from the periportal areas in which the prevailing cellular alteration is proliferation of mitochondria (Bannasch 1996). Interestingly, Nakajima et al. (2000) also reported that TCE induced peroxisomes in perivenular but not in periportal areas of mice liver.

One study showed that extraperoxisomal effects of PPARα agonists that may be related to tumor induction are effects on mitochondria, which have a role in several aspects of tumor biology and whose DNA may have increased susceptibility. Zhou and Wallace (1999) reported that GEM and WY induced the mitochondrial permeability transition as characterized by calcium-induced swelling and depolarization of membrane potential, both of which were inhibited by cyclosporine A. Fenofibrate, clofibrate, ciprofibrate, and DEHP, on the other hand, caused a direct dose-dependent depolarization of mitochondrial membrane potential. However, the mechanism of membrane depolarization varied among the test chemicals. Bezafibrate and TCE elicited no effect on succinate-supported mitochondrial bioenergetics. The authors concluded that most but not all the peroxisome proliferators they studied interfered with mitochondrial bioenergetics and that the specific biomolecular mechanism differed among the individual compounds. Peroxisome proliferators have also been reported to induce pronounced mitochondrial proliferation and increased activity of mitochondrial enzymes in liver tumors (Bannasch et al. 2001).

Polyak et al. (1998) have examined mitochondria and neoplasia, primarily because of their role in apoptosis and other aspects of tumor biology. The mitochondrial genome is particularly susceptible to mutations because of the high level of reactive oxygen species generation in this organelle coupled with a low level of DNA repair. The authors reported mutations in the mitochondrial genome in most human colorectal cancers examined. Petros et al. (2005) reported mutations in the mitochondrial DNA (mtDNA) that have been found to fulfill all the criteria expected for pathogenic mutations causing prostate cancer. Booker et al. (2006) reported that the highly polymorphic mitochondrial genome, which is separate from nuclear DNA, confers an inherited cancer risk for prostate and renal cancers. Possible effects of peroxisome proliferators on mitochondrial genomics have not been investigated.

Another area of active investigation has been whether PPARα agonists activate nonparenchymal liver cells such as Kupffer cells independently of PPARα activation and whether such activation may be necessary for tumor induction, particularly due to their role in parenchymal cell proliferation and apoptosis suppression (Hasmall et al. 2001; Holden et al. 2000; Parzefall et al. 2001; Peters et al. 2000; Roberts et al. 2002; Rusyn et al. 2000, 2001). Although the hypothesized MOA for induction of acyl–CoA oxidase (ACO) leading to increased production of H_2_O_2_ and DNA damage seems unlikely, free radicals may be important in signaling Kupffer cells to produce mitogenic cytokines [e.g., tumor necrosis factor α (TNF-α)].

Rusyn et al. (2000, 2001) suggest that cell proliferation and tumors require parenchymal cell PPARα and TNF-α production by Kupffer cells. They also suggest that peroxisome proliferators increase free radicals in the liver before peroxisomal oxidases are induced and activate the transcription factor nuclear factor κB (NF-κB; one of the major regulators of TNF-α expression) in Kupffer cells. Interestingly, they report that corn oil (often used as a vehicle) rapidly activated NF-κB in Kupffer cells and triggered production of low levels of TNF-α. Other studies support TNF-α acting downstream or independently of PPARα to mediate the suppression of apoptosis and induction of DNA synthesis by peroxisome proliferators (Holden et al. 2000; Peters et al. 2000; Roberts et al. 2002). Klaunig et al. (2003) noted that responsiveness (or lack thereof) in human hepatocyte assay systems could be linked to removal of Kupffer cells during preparation.

## Pleiotropic Responses and Actions of PPARα

Although studies of TCA, DCA, and other PPARα agonists in human hepatocyte cultures seem to indicate that the human liver is refractory to markers of PP (e.g., Walgren et al. 2000a, 2000b), humans are responsive to at least some other effects from PPARα agonism, as evidenced by the efficacy of hypolipidemic fibrate drugs. An extensive research effort into PPARs, much of it published since 2001, has been set off by evidence that highly prevalent chronic diseases such as diabetes, obesity, atherosclerosis, and cancer may involve PPAR activity and may be affected by PPAR agonists such as thiazolidinediones and fibrates (Kersten et al. 2000). Table 1 summarizes some of the recent literature regarding activities and effect of activation of the PPARα receptor, demonstrating its pleiotropic nature. Along with the liver, other target organs and systems affected include muscle, cardiovascular system, small intestine, testes, ovary, thyroid, adrenal axis, and immune system.

In the liver, PPARα responses involve not only the parenchymal cells of the liver (hepatocytes), but also macrophages (Kupffer cells). Activities affected include lipid and glucose metabolism; bile acid synthesis; macrophage cholesterol homeostasis, inflammatory cytokine production, and recruitment to inflammatory sites; actions and control of hormones (glucocorticoids, growth hormones thyroid estrogen); and protein expression (those involved with all stages of atherosclerosis, liver fatty acid binding, male rat-specific α_2_μ-globulin, a mouse homologue of α_2_μ, glutathione *S*-transferase, glutathione reductase, and the CYP genes *cyp2b*, *cyp2c*, *cyp3a*, *cyp1a1*, and *cyp4a*). Effects on the vulnerability of the liver to other insults such as acetaminophen toxicity have also been reported. Moreover, because some of these extraperoxisomal effects of PPARα agonists may not depend on interaction with PPARα, Scatena et al. (2003) suggest that the biochemical profile and a therapeutic role of this class of PPAR ligands are more complex than previously proposed.

That PPARα agonism results in pleiotropic responses should not be surprising. Poole et al. (2001) have shown that after an agonist binds to the PPARα receptor, it heterodimerizes with the retinoid X receptor, with the heterodimer interacting with DNA sequences or response elements found in a large number of responsive genes. An examination of the full spectrum of PPARα activity is necessary to make a comprehensive comparison with TCE-induced effects, and a number of issues in examining and interpreting these data are discussed in the sections that follow.

### Gene regulation and expression

There is a growing database on the differences in responses among PPARα agonists as well as the pleiotropic responses they induce. Some agonists have been shown to display activity toward more than one receptor (Berger and Moller 2002; Liu et al. 2005), which complicates interpretation of data across chemicals. Using the same paradigm, an examination of several recent publications, summarized in Table 2, reveals inconsistent results between PPARα agonists, paradoxes between mRNA and protein expression, strain, gender, and species differences in response to the same chemical, and time-dependent differences in response (Fan et al. 2003, 2004; O’Brien et al. 2001; Poole et al. 2001).

In addition male rats have been reported to be more responsive to fibrates than are female rats. Jalouli et al. (2003) reported that male rats had higher levels of hepatic PPARα mRNA and protein than did female rats. The authors suggested that sex hormones regulate the sex difference in hepatic PPARα levels but not via the sexually dimorphic growth hormone secretory pattern. Nakajima et al. (2000) reported no remarkable sex difference in TCE-induced PP in wild-type mice, as measured morphologically, but a markedly higher induction of several enzymes and PPARα protein and mRNA was found in the liver of males after 2 weeks of exposure.

As mentioned above, *PPAR*α knockout mice have been used to make inferences about PPARα expression effects, but no common pattern of gene expression has emerged. Valles et al. (2003) reported exposure of diisononyl phthalate in B6C3F_1_ and SV129 wild-type and knockout mice to show a varied pattern of gene expression dependent on gender and age. They suggested that some changes in gene expression were dependent on PPARα activity and others were not. Macdonald et al. (2001) reported alteration of 59 PPARα- and peroxisome-dependent proteins after DEHP treatment. Proteins identified as being regulated by PPARα were known to be involved not only in lipid metabolism pathways but also in amino acid and carbohydrate metabolism, mitochondrial bioenergetics, and stress responses, including several genes not previously reported to be regulated by PPARα. Hasmall et al. (2002) reported a 3- to 7-fold down-regulation of lactoferrin mRNA in response to DEHP in wild-type versus *PPAR*α knockout mice. The authors suggested that the regulation of iron-binding proteins by PPARα ligands plays a role in peroxisome proliferator–mediated liver growth but not in PP.

Another approach for investigation of PPARα related effects is to study its overex-pression. Jia et al. (2003) reported that disruption of the inducible β-oxidation pathway in mice at the level of fatty ACO results in spontaneous PP and sustained activation of PPARα. Meyer et al. (2003) used cDNA microarrays to study the expression profiles of 26 hepatocellular carcinomas developing spontaneously in peroxisomal fatty ACO knockout mice. Comparisons of the knockout mouse liver tumor expression profiles with those induced by ciprofibrate or diethylnitrosamine showed that these mice shared a number of deregulated (up- or down-regulated) genes with ciprofibrate-induced liver tumors.

### Use of PPARα knockout mice to study MOA

Several studies have used *PPAR*α knockout mice to try to determine specific responses associated with PPAR agonism and potential MOA of liver cancer induction, but concerns have been raised regarding the adequacy of this model. These are related to both existing study designs (e.g., a less-than-lifetime analysis of tumor induction) and to whether the intrinsic characteristics of these knockout mice mean that they exhibit responses that differ from those of wild-type mice independent of effects related to PPARα agonism. The recent study by Laughter et al. (2004), discussed above, illustrates the potential difficulties in interpreting studies using knockout mice.

Huss and Kelly (2004) reported massive cardiac lipid accumulation and hepatic steatosis in *PPAR*α knockout mice after fasting or pharmacologic inhibition of fatty acid oxidation. Such mice have reduced cardiac expression of genes involved in the cellular uptake, mitochondrial transport, and mitochondrial (and peroxisomal) oxidation of fatty acids. After exposure to stress, *PPAR*α knockout mice have decreased ATP concentration with abnormal cristae of the mitochondria, abnormal caveolae, and fibrosis in the myocardium in an age-dependent manner (Watanabe et al. 2000). After partial hepatectomy, *PPAR*α knockout mice have a 12- to 24-hr delay in liver regeneration and hepatic gene expression with a delayed onset and lower peak magnitude of hepatocellular DNA synthesis (Anderson et al. 2002). Furthermore, these mice had a 24-hr lag in the hepatic expression of the G_1_/S checkpoint regulator genes cyclin D1 (*Ccnd1*) and *c-myc* and increased expression of the interleukin-1β cytokine gene (genes involved in cell cycle control, cytokine signaling, and fat metabolism). Epidermal regeneration has also been reported to be affected in *PPAR*α knockout mice (Michalik et al. 2001, 2002).

Costet et al. (1998) reported that with stable caloric intake, *PPAR*α knockout mice were a model of monogenic, spontaneous, late-onset obesity, with a marked sexual dimorphism. Increased serum triglycerides, cholesterol, and phosholipids were elevated in aged *PPAR*α knockout mice, with higher serum triglycerides in females. Females also developed a more pronounced obesity than did males but no steatosis. Males showed a marked steatosis restricted to the centrilobular region, a delayed occurrence of obesity, and larger elevation in hepatic cholesterol and triglycerides than did females or wild-type mice. By 302 days, normal hepatocytes were restricted to periportal zones. All animals showed an increase in all fat tissues (including brown fat).

Shankar et al. (2003) also reported *PPAR*α knockout mice to have significant steatosis without treatment. Lewitt et al. (2001) reported *PPAR*α knockout mice to have a sexually dimorphic phenotype, with PPARα influencing the IGF/IGF-binding protein response to feeding, particularly in males, and suggested that gender differences in the IGF system contribute to the *PPAR*α knockout phenotype. It has been suggested that elevated serum levels of IGF1 and leptin are associated with increased risk of developing cancer (Hursting et al. 2003; Liu et al. 2001; Sandhu et al. 2002; Thompson et al. 1999). Not only are hepatocytes abnormal and adversely affected from knockout of the *PPAR*α gene, but full expression of carcinogenicity, especially by weaker agonists, may be limited by decreased survival [i.e., untreated knockout mice begin to die by age 3 months, with a 50% mortality rate by 6 months and 100% mortality rate by 11 months of age (Nohammer et al. 2003)].

### Intrinsic factors that may affect PPAR-mediated risks

Important considerations in trying to determine the potential effects of PPAR agonists and how they may contribute to TCE toxicity and risk are the intrinsic factors that affect that risk. Modulation of PPAR-mediated risks by intrinsic factors such as genetic polymorphisms, disease states, and life stages may give important clues about key steps in their MOAs and the effects of agonism or changes in receptor function. A number of recent studies are summarized below that are representative of the issues currently under investigation. Although a definitive picture has yet to emerge, the investigations of polymorphic responses in particular could be informative of potential human uncertainty and variability in susceptibility to a number of end points and targets besides the liver.

Graham et al. (2004) recently reported significantly increased incidence of hospitalized rhabdomyolysis in patients treated with fibrates both alone and in combination with statins. Brisson et al. (2002) suggest that frequent genetic variations in genes encoding proteins involved in triglyceride-rich lipoprotein metabolism could modulate the response to fenofibrate treatment, as defined in clinical guidelines. Robitaille et al. (2004) reported that the *PPAR*α-*L162V* polymorphism alone or in interaction with dietary fat intake was associated with components of the metabolic syndrome. Vohl et al. (2000) reported an association between the *PPAR*α *V162* allele and the atherogenic/hyperapolipoprotein B dyslipidemia. Jamshidi et al. (2002) reported that variation in the *PPAR*α gene influenced human left ventricular growth in response to exercise and hypertension, indicating that maladaptive cardiac substrate use can play a causative role in the pathogenesis of left ventricular hypertrophy. Eurlings et al. (2002) reported that the *PPAR*α gene was a modifier of the familial combined hyperlipidemia phenotype [a common genetic lipid disorder present in 10% of patients with premature coronary artery disease (CAD)]. Lacquemant et al. (2000) screened the *PPAR*α gene for mutations to test the genetic contribution of the PPARα in diabetes and its vascular complications and concluded that it is unlikely that the *PPAR*α gene had a major role in diabetes and CAD in their populations.

Huss and Kelly (2004) suggested that PPARα and PPARβ are primary regulators of fatty acid metabolism in the heart and that disturbances of PPARα either through inactivation or chronic stimulation can have deleterious effects, particularly in the context of diabetes, hyperlipidemic states, or the ischemic heart. The insulin-resistant and diabetic heart is characterized by increased fatty acid oxidation rates that may be related to chronic stimulation of the *PPAR*α gene regulatory pathway. Mice genetically modified to mimic the metabolic derangements of the diabetic heart (i.e., cardiac-specific overexpression of PPARα) (Harris et al. 2004) had ventricular diastolic/systolic dysfunction at baseline, which was exacerbated by high-fat feeding or insulinopenia, and developed cardiomyopathy. Jove et al. (2004) reported that decrease of mtDNA content has been related to the pathogenesis of type 2 diabetes mellitus and showed increased expression of PPARα and its target genes to be involved in fatty acid metabolism in skeletal muscle of Zucker diabetic fatty rats. Asayama et al. (1999) have reported PPARα expression and activity to be increased in diabetic rat liver.

Regarding life stages, there is also evidence that peroxisome proliferators are much more potent in producing tumors in older rats than in younger ones, even though effects on PP and cell proliferation were the same (Chao et al. 2002; Youssef and Badr 2002; Youssef et al. 2003). A promotion effect in older animals with already initiated foci could be the MOA for increased sensitivity of older rats to PPARα effects. Specific time- and tissue-dependent patterns of PPARα, PPARδ, and PPARγ expression have been shown during fetal development and in adult animals (Michalik et al. 2001, 2002). Data on humans are limited. Other factors in the developing rodent or human (i.e., differences in cell proliferation, xenobiotic metabolism) could affect sensitivity to PPARα hepatocarcinogenesis. Ring et al. (1999) reported that in addition to differences in metabolic enzymes, the fetal liver has a unique physiologic milieu (e.g., fetal hepatic circulation and zonation of drug-metabolizing enzymes along the hepatic acinus differs substantially from the adult). Placental transfer of the clofibrate with increased PP and CYP4A mRNA has been demonstrated in both maternal and fetal livers (3-fold mRNA elevation in fetuses) (Simpson et al. 1996), as has translactational induction of CYP4A expression by clofibrate in neonatal rats (Simpson et al. 1995).

## Summary

The studies reviewed here suggest that, given its pleiotropic responses, PPARα agonism may play a complex role in cell signaling and gene expression changes that contribute to a variety of different diseases and effects. Unfortunately, common patterns of gene expression changes among TCE, its metabolites, and other PPARα agonists, particularly those related to tumorigenic responses, have yet to be identified, precluding their use in delineating common MOAs. Recent data also suggest that even for liver tumor induction, extraperoxisomal effects such as changes in mitochondria and activation of Kupffer cells may play an important role, so inferences based on PP or purified hepatocyte cultures alone may be misleading. Recent studies also suggest that knockout and wild-type mice have baseline differences in liver parameters before treatment and exhibit differences in response to agonists, including TCE and its metabolites, independent of the peroxisomal effects, making interpretation of such studies challenging. On the whole, recent studies suggest that inferences regarding the MOA(s)—and hence the human relevance and susceptibility—of TCE-induced effects require a better understanding of the interplay of extraperoxisomal events after PPARα agonism.

## Figures and Tables

**Table 1:** Recent literature on effects associated with PPARα agonism or related to its mechanisms of action.

Effect	Reference
Role in chronic diseases: obesity, atherosclerosis, diabetes, inflammation, and cancer	Barbier et al. (2002), Berger and Moller (2002), Berger and Wagner (2002), Guerre-Millo et al. (2001), Hays et al. (2005), Jove et al. (2004), Kersten et al. (2000), Lacquemant et al. (2000), Liu et al. (2005), Moennikes et al. (2003), Moller and Berger (2003), Robitaille et al. (2004), Shankar et al. (2003), Vohl et al. (2000), Vosper et al. (2002)
Role in fasting	Escher et al. (2001), Kersten et al. (2001), Poirier et al. (2001)
Changes in susceptibility to disease: cardiomyopathies and cardiac cell metabolism, familial combined hyperlipidemia, increased susceptibility from aging, and acetaminophen hepatotoxicity	Brisson et al. (2002), Chao et al. (2002), Chen et al. (2000, 2002), Eurlings et al. (2002), Harris et al. (2004), Huss and Kelly (2005), Huss et al. (2005), Jamshidi et al. (2002), Jiang et al. (2004), Nohammer et al. (2003), Watanabe et al. (2000), Youssef and Badr (2002), Youssef et al. (2003)
Extrahepatic effects: muscle lipid homeostasis, liver fatty acid–binding protein (liver and small intestine), and early inflammation phase of the healing	Michalik et al. (2001), Muoio et al. (2002), Poirier et al. (2001)
Cell signaling effects: TNF-α, growth hormone and STAT5b, L-pyruvate kinase (glycolytic enzyme), and bile acid synthesis and catabolism in the liver (UDP-glucuronosyltransferase)	Barbier et al. (2003a, 2003b), Holden et al. (2000), Pan et al. (2000), Peters et al. (2000), Roberts et al. (2002), Rusyn et al. (2000), Sinal et al. (2001), Zhou and Waxman (1999), Zhou et al. (2002)
Phase I and II enzymes—CYP expression changes: CYP genes (including CYP2B, CYP2C, CYP3A, CYP1A1, and CYP4A family members), modulation of glutathione defense	Fan et al. (2003, 2004), Kim et al. (2003), O’Brien et al. (2001), Ripp et al. (2003), Seree et al. (2004)
Endocrine effects: ovarian function, estrogen action, steroid metabolism enzymes, testicular degeneration, and thyroid hormone action	Dufour et al. (2003), Gazouli et al. (2002), Kim et al. (2003), Klotz et al. (2000), Komar et al. (2001), Miller et al. (2001), Parks et al. (2000), Poole et al. (2001), Xu et al. (2001), Zhu et al. (1999)

**Table 2:** Examples of chemical-, gender-, species-, and PPARα polymorphism-dependent responses to PPARα agonists.^a^

Parameter	Test subjects	WY	DBP	GEM	DEHP
NADPH–CYP oxidoreductase					
mRNA	F-344 male rat	↑ 4.4-fold	↑ 2.2-fold	No change	—
	F-344 female rat	↑ 7.2-fold	↑ 5.1-fold	↑ 4.4-fold	—
	Wild-type male mouse	↑ 4.6-fold	—	—	↑ 5.8-fold
	PPARα null male mouse	No change	—	—	No change
Protein	F-344 male rat	↓ to 29%	No change	↓ to 18%	—
	F-344 female rat	No change	↑ 3.2-fold	No change	—
	SD male rat	↓ to 40%	—	↓ to 14%	—
	Wild-type male mouse	↓ to 4%	—	—	↓ to 12%
	PPARα null male mouse	No change	—	—	↑ 2.0-fold
Nonspecific carboxyesterase protein^b^					
ES-4	F-344 male rat	↓ to 30%	No change	↓ to 15%	—
	F-344 female rat	No change	No change	↑ 1.6-fold	—
	SD male rat (#1)	↓ to 12%	↓ to 39%	↓ to 32%	—
	SD male rat (#2)	↓ to 13%	↓ to 63%	↓ to 16%	—
	Wild-type male mouse	No change	—	—	No change
	PPARα male null mouse	No change	—	—	No change
ES-10	F-344 male rat	↓ to 1%	No change	↓ to 10%	—
	F-344 female rat	↓ to 10%	↑ 2.0-fold	No change	—
	SD male rat (#1)	↓ to 7%	↓ to 59%	↓ to 16%	—
	SD male rat (#2)	↓ to 8%	↓ to 60%	↑ 1.4-fold	—
	Wild-type male mouse	No change	—	—	No change
	PPARα null male mouse	No change	—	—	↓ to 50%
2α-Testosterone hydroxylase activity	F-344 male rat	↓ to < 1%	↓ to 43%	↓ to 31%	—
6β-Testosterone hydroxylase activity	F-344 male rat	No change	↑ 2.6-fold	↑ 2.0-fold	—
7α-Testosterone hydroxylase activity	F-344 male rat	No change	No change	No change	—
16α-Testosterone hydroxylase activity	F-344 male rat	↓ to 4%	↓ to 47%	↓ to 35%	—
16β-Testosterone hydroxylase activity	F-344 male rat	↑ 2.3-fold	↑ 3.2-fold	↑ 3.6-fold	—
Androstenedione hydroxylase activity	F-344 male rat	↓ to 24%	No change	No change	—
CYP3A11 mRNA (6α-testoserone hydroxylase)	Wild-type male mouse	↓ to 40%	—	—	↑ 5.7-fold
	PPARα null male mouse	↑ 1.9-fold	—	—	↑ 5.7-fold
CYP3A2 mRNA	F-344 male rat	↓ to 25%	No change	↓ to 36%	—
CYP3A2 protein^b^	F-344 male rat	↓ to 13%	↑ 1.9-fold	No change	—
	F-344 female rat	No change	↑ 5.0-fold	↑ 5.0-fold	—
	SD male rat (#1)	↓ to 15%	↓ to 57%	No change	—
	SD male rat (#2)	↓ to 3%	No change	No change	—
CYP3A1 protein	F-344 male rat	↑ 11-fold	↑ 15-fold	↑ 2-fold	—
	F-344 female rat	↓ to 42%	↑ 4.6-fold	↓ to 50%	—
CYP2B1 protein	F-344 male rat	No change	↑ 2.4-fold	No change	—
	F-344 female rat	No change	↑ 8.0-fold	↑ 3.9-fold	—
CYP4A protein	F-344 male rat	↑ > 80-fold	↑ > 60-fold	↑ > 16-fold	—
	F-344 female rat	↑ 60-fold	No change	No change	—
Estrogen sulfotransferase protein	F-344 male rat	↓ to 2%	↓ to 8%	↓ to12%	—
	F-344 female rat^c^	↓	↓	↓	—
Glutathione *S*-transferase^d^	SD male rat	↓ to 11%	↓ to 43%	No change	—
Selenium-dependent glutathione peroxidase^d^	SD male rat	↓ to 66%	↓ to 76%	No change	—
Glutathione equivalents^d^	SD male rat	No change	↓ to 66%	No change	—

Abbreviations: —, not tested; ↑, increased; ↓, decreased; DBP, dibutyl phthalate; SD, Sprague-Dawley.

a Results are from Poole et al. (2001), Fan et al. (2003, 2004), and O’Brien et al. (2001) in which F-344 rats, Sprague-Dawley rats, or SV129 PPARα (+/+) or (−/−) “null” or “knockout” mice were exposed for 13 (rats) or 3 (mice) weeks. Rats received control diet, 500 ppm WY, 8,000 ppm GEM, or 20,000 ppm dibutyl phthalate in the diet. Mice received control diet, 0.1% WY, or 0.6% DEHP in diet.

b Results from Fan et al. (2004) and Poole et al. (2001) included two sets of experiments for Sprague-Dawley rats.

c No quantitative number given but reported to be statistically significant. Testosterone hydroxylase activities are derived from hepatic microsomes.

d Exposure level of GEM is 16,000 ppm. Parameters investigated in the liver include NADPH–CYP oxidoreductase, an often rate-limiting component in CYP-dependent reactions; nonspecific carboxyesterases, a large group of enzymes that play important roles in the metabolism of endogenous lipids and foreign compounds such as pesticides and drugs; phase I and II steroid metabolism enzymes; and glutathione and glutathione-related enzyme activities.
